# Immunohistochemical analysis of PDK1 expression in breast cancer

**DOI:** 10.1186/1746-1596-9-82

**Published:** 2014-04-16

**Authors:** Ruza Arsenic

**Affiliations:** 1 Institute of Pathology Charité, University Hospital Berlin, 10117 Berlin, Germany

**Keywords:** PDK1, PIK3CA, Breast cancer

## Abstract

**Background:**

3-phosphoinositide-dependent protein kinase-1 (PDK1) functions downstream of phosphoinositide 3-kinase (PIK3) and activates members of the AGC family of protein kinases that are known to play crucial roles in physiological processes associated with cell metabolism, growth, proliferation and survival. Changes in the expression and activity of PDK1 and several AGC kinases have been linked to human disease, including cancer.

**Methods:**

We used immunohistochemical analysis to determine PDK1 expression in 241 tumors from patients with breast cancer in which we had previously analyzed *PIK3CA* mutation status.

**Results:**

Moderate or high expression of PDK1 was observed in 213 of the 241 cases (88%). There was no correlation between *PIK3CA* mutation status and PDK1 overexpression.

**Conclusion:**

Our findings indicate that PDK1 is independently activated in breast cancer and not only as part of the PIK3CA pathway, suggesting that PDK1 plays a specific and distinct role from the canonical PIK3/Akt pathway and promotes oncogenesis independently of AKT. Our data implicate PDK-1 and downstream components of the PDK-1 signaling pathway as promising therapeutic targets for the treatment of breast cancer.

## 

The protein kinase 3-phosphoinositide-dependent protein kinase-1 (PDK1) plays a key role in signaling pathways activated by several growth factors and hormones. PDK1 functions downstream of phosphoinositide 3-kinase (PIK3) and activates members of the AGC family of protein kinases such as protein kinase B (Akt), protein kinase C (PKC), p70 ribosomal protein S6 kinases, and serum glucocorticoid-dependent kinase by phosphorylating serine/threonine residues in the activation loop. AGC kinases are known to play crucial roles in the regulation of various physiological processes relevant to metabolism, growth, proliferation, and survival.

The first evidence that PDK1 might be a viable target in cancer came in 2005 when Bayascas et al. [1] generated transgenic mice that were hypomorphic for PDK1. These mice were crossed with tumorigenic heterozygous *PTEN +/-* mice and the resulting mice with deficient PDK1 levels had a reduced prevalence of tumor development. Subsequent studies demonstrated the role of PDK1 in a variety of different cancers; in particular PDK1 appears to play a decisive role in the development of breast cancer [2].

Increased PDK1 expression has also been reported in 45% of patients with acute myeloid leukemia, and PDK1 seems to be a viable target in head and neck cancer, multiple myeloma, pancreatic cancer, and colorectal cancer [3-7]. Vasudevan et al. [8] reported that a subset of breast cancer cell lines with mutations in *PIK3CA* displayed a reduced dependence on Akt for tumorigenicity, and instead relied on PDK1-dependent activation of another AGC kinase, SGK-3. Breast cancers are known to be a group of diverse diseases, and cellular heterogeneity has been shown to affect disease-free survival in patients with breast cancer [9]. For example, expression of CARM1 varies widely among different molecular subtypes of breast cancer, and overexpression of CARM1 is associated with invasive cancer and a poor prognosis [10]. Molecules such as CARM1 may have potential clinical applications in prognostic stratification and therapeutic molecular targeting.

There is accumulating evidence that PDK1 is overexpressed in particular cancer settings and activates cancer cell growth and survival independent of Akt signaling. These findings suggest that PDK1 is not just an Akt-activating player, but rather an important oncogenetic regulator and a potential therapeutic target in cancer. Recently, it has been shown that PDK1 regulates anchorage-independent growth, resistance to several anticancer drugs, and tumor formation in breast cancer cells–not only in tumors harboring *PIK3CA* mutations, but also in the absence of these genetic alterations [11].

This study aimed to answer the following questions: (1) Is PDK1 overexpressed in breast cancer and to what extent? (2) Is there any correlation between PDK1 overexpression and *PIK3CA* mutations?

To address these questions, we examined the phosphorylation status of PDK1 in a group of tumors in which we had previously analyzed *PIK3CA* mutations [12]. Four tissue microarrays were generated from formalin-fixed and paraffin-embedded (FFPE) specimens using a precision instrument (Beecher instruments, Silver Spring, MD, USA). A representative tumor-bearing slide was selected for each case by a board certified pathologist with a special interest in breast pathology (RA). Typical tumor areas were marked on the respective H&E slides. Subsequently two tissue cylinders of 1.5 mm diameter were punched from each tumor-bearing donor block and transferred to a tissue microarray paraffin block. For immunohistochemistry, 3-μm paraffin sections were cut and incubated with antibody to pPDK1. Omission of the primary antibody served as the negative control. The primary antibody used in this study was purchased from Cell Signaling Technology Inc. (Beverly, MA) and was highly validated by the manufacturer (pPDK1, S241, 1 : 50 dilution). The overall intensity of immunohistochemical staining on ductal and lobular regions within each tissue was scored visually and graded as follows: 0, negative staining; 1, weak staining; 2, moderate staining; and 3, intensive staining. A score of 2 or greater was required for a tissue to be classified as positive for phosphorylation. Representative images showing staining patterns corresponding to each score are shown in Figure 1.

**Figure 1:**
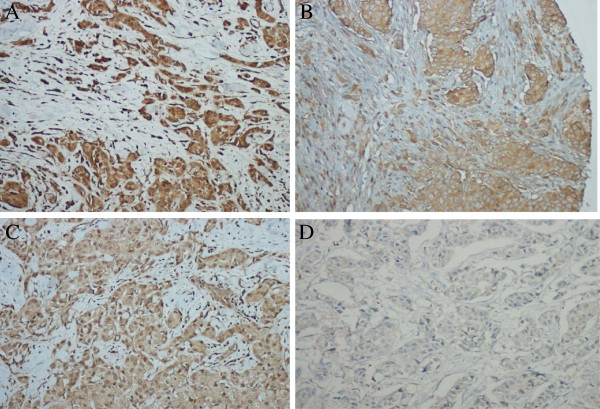
**Immunohistochemical expression of PDK1 (3-phosphoinositide-dependent protein kinase-1) in breast cancer. A**-tumor cells with strong immunostaining, score 3, **B**-tumor cells with moderate immunostaining-score2, **C**-tumor cells with weak immunostaining, score 1, **D**-tumor cells negative, score 0.

Moderate or high expression of PDK1 was observed in 213 of the 241 cases (88%). *PIK3CA* mutations were identified in 15.8% of cases in the same patient collective. There was no correlation between *PIK3CA* mutation status and PDK1 overexpression.

The fact that some cases without PIK3CA mutations had a moderate or high expression of PDK1 suggests that PDK1 can be independently activated in breast cancer and not only as part of the PIK3CA pathway. Our results indicate that PDK1 plays a specific role distinct from the canonical PIK3/Akt pathway.

To the best of our knowledge this study is the first to compare *PIK3CA* mutation status and PDK1 expression in tissues from the same tumor group. The results of our study suggest that the PDK-1 signaling pathway might be a promising therapeutic target for the treatment of breast cancer and that PDK1 expression should be tested in addition to *PIK3CA* status in patients with breast cancer prior to beginning therapy.

## Abbreviations

PDK1: 3-phosphoinositide-dependent protein kinase-1; PIK3CA: phosphatidylinositol-4,5-bisphosphate 3-kinase, catalytic subunit alpha.

## Competing interests

The authors declare that they have no competing interest.
